# Clinical outcomes of breast-conserving surgery versus mastectomy in early-stage breast cancer: A retrospective cohort study

**DOI:** 10.6026/973206300213202

**Published:** 2025-09-30

**Authors:** Rohit Dubey, Ambuj Kumar Soni, Vinit Kumar Tiwari, Rajkishor Singh

**Affiliations:** 1 Department of Surgery, Government Medical College, Satna, Madhya Pradesh, India; 2 Department of Surgery, Birsa Munda Government Medical College, Shahdol, Madhya Pradesh, India; 3 Department of Surgery, Atal Bihari Vajpayee Government Medical College, Vidisha, Madhya Pradesh, India

**Keywords:** Breast-conserving surgery, mastectomy, early-stage breast cancer, survival outcomes, recurrence rates

## Abstract

The best surgical approach for early-stage breast cancer is still under debate, particularly in the context of breast-conserving surgery (BCS) and mastectomy. A retrospective cohort study of 132 patients treated with BCS or mastectomy over 5 years of follow-up compared outcomes related to overall survival, disease-free survival, recurrence, and complications. Outcomes were similar in each group but higher recurrence was associated with BCS and better cosmetic and psychological outcomes. Mastectomy had lower recurrence but additional morbidity associated with treatment. These findings are an important step in advocating for shared decision-making with individualized treatment options.

## Background:

Breast cancer remains the most common malignancy among women globally, with early-stage breast cancer comprising a significant proportion of diagnoses due to increased awareness and widespread screening programs [1]. Historically, mastectomy was considered the gold standard for surgical management [2]. However, breast-conserving surgery (BCS), combined with radiotherapy, has emerged as an effective alternative offering equivalent survival outcomes in select patient populations [3]. Several randomized controlled trials and long-term observational studies have demonstrated comparable overall and disease-free survival between BCS and mastectomy in early-stage breast cancer [4]. Despite this, surgical decision-making is often influenced by patient preference, surgeon recommendation, tumor characteristics, access to radiotherapy and socio-cultural factors [5]. Concerns about recurrence, cosmetic outcomes, body image and psychological health further complicate the choice of treatment [6]. Therefore, it is of interest to compare the clinical outcomes of breast-conserving surgery versus mastectomy in early-stage breast cancer to inform patient-centered decision-making in real-world settings.

## Materials and Methods:

This retrospective cohort study was conducted at a tertiary care hospital and included 132 female patients diagnosed with early-stage (Stage I and II) breast cancer between January 2017 and December 2021. Patient data were retrieved from electronic medical records and operative databases. Eligible participants were those who underwent either breast-conserving surgery (BCS) followed by radiotherapy or total mastectomy without prior neo adjuvant therapy. Patients with metastatic disease at diagnosis, previous malignancy, or incomplete records were excluded from the analysis. Patients were divided into two groups based on the type of surgery received: BCS group (n=68) and mastectomy group (n=64). Clinical and pathological variables including age, tumor size, lymph node status, histological subtype, hormone receptor status and HER2 status were collected. Outcomes analyzed included overall survival, disease-free survival, local recurrence, distant metastasis and post-operative complications over a follow-up period of up to 5 years. Statistical analysis was performed using SPSS software. Kaplan-Meier curves were used to estimate survival outcomes and chi-square and t-tests were used for group comparisons, with a p-value <0.05 considered statistically significant.

## Results:

A total of 132 patients were analyzed, with 68 undergoing breast-conserving surgery (BCS) and 64 undergoing mastectomies. Both groups were comparable in baseline characteristics. While overall survival and disease-free survival were similar between groups, the BCS group reported better psychological and cosmetic outcomes, though local recurrence was slightly higher. Table 1 (see PDF) outlines the demographic characteristics of the study population. Patients undergoing breast-conserving surgery (BCS) and mastectomy were similar in terms of mean age, menopausal status and family history of breast cancer, indicating well-balanced cohorts for comparative analysis. Table 2 (see PDF) presents the tumor characteristics, showing no significant differences between groups with respect to tumor stage, histological subtype (predominantly invasive ductal carcinoma), tumor grade and hormone receptor/HER2 status. This uniformity supports the validity of outcome comparisons. Table 3 (see PDF) highlights surgical margin status postoperatively. Positive margins were significantly more frequent in the BCS group (11.8%), necessitating re-excisions in some cases, while all mastectomy patients had clear margins, reflecting the more extensive nature of the procedure. Table 4 (see PDF) summarizes the distribution of adjuvant therapies. While chemotherapy and hormonal therapy usage were similar across both groups, radiotherapy was universally administered in the BCS group but only selectively in the mastectomy group, as expected by standard care protocols. Table 5 (see PDF) compares recurrence and metastasis outcomes. Local recurrence occurred more frequently in the BCS group (8.8%) than in the mastectomy group (3.1%), though this difference was not statistically significant. Distant metastasis rates were nearly identical between groups. Table 6 (see PDF) details five-year overall survival rates. Survival outcomes were nearly equivalent between the BCS and mastectomy groups, with over 90% of patients alive in both cohorts, reinforcing the oncologic equivalency of both surgical options. Table 7 (see PDF) assesses five-year disease-free survival. Slightly more recurrences were observed in the BCS group (14.7%) compared to the mastectomy group (12.5%), although the difference was not statistically significant, confirming the long-term efficacy of BCS. Table 8 (see PDF) captures patient-reported outcomes on cosmetic and psychological well-being. Patients in the BCS group reported significantly greater satisfaction with appearance, a more positive body image and better psychological health, emphasizing the quality-of-life advantages of breast conservation. Table 9 (see PDF) evaluates postoperative complications. Mastectomy was associated with a higher, albeit non-significant, incidence of seroma formation, surgical site infection and delayed wound healing compared to BCS, consistent with the larger extent of surgery. Table 10 (see PDF) examines hospital stay duration. The BCS group had a significantly shorter average hospital stay (2.4 days) compared to the mastectomy group (4.8 days), indicating quicker recovery and potentially lower healthcare resource utilization with breast-conserving approaches.

## Discussion:

This retrospective cohort study compared clinical outcomes between breast-conserving surgery (BCS) and mastectomy in patients with early-stage breast cancer. The findings reaffirm that BCS, when followed by radiotherapy, offers overall and disease-free survival outcomes comparable to mastectomy. Although local recurrence was slightly higher in the BCS group, it did not translate into reduced overall survival, underscoring the oncologic safety of breast conservation in appropriately selected patients [7]. Psychosocial and cosmetic advantages were significantly greater in the BCS group, contributing to higher patient satisfaction and better body image [8]. These benefits are particularly important in younger, premenopausal patients where quality of life plays a central role in surgical decision-making. On the other hand, mastectomy was associated with fewer positive surgical margins but had a marginally higher incidence of post-operative complications such as infection and seroma formation, likely due to the more extensive nature of the procedure [9, 10]. The length of hospital stay and time to return to daily activities were also shorter in the BCS group, suggesting potential cost-effectiveness and quicker rehabilitation [11]. These practical outcomes, combined with similar long-term survival, support the growing preference for BCS in early-stage breast cancer where technically feasible [12]. The main limitation of the study lies in its retrospective design and single-center setting, which may limit generalizability [13]. Nevertheless, these real-world findings highlight the importance of shared decision-making and patient-centered care in surgical planning for breast cancer.

## Conclusion:

Breast-conserving surgery offers survival outcomes comparable to mastectomy in early-stage breast cancer, with added benefits in cosmetic satisfaction and psychological well-being. Although local recurrence is slightly higher with BCS, it does not affect overall prognosis. Surgical decisions should be individualized, balancing oncologic safety with patient preferences and quality of life.

## References

[1] Rajan KK (2024). BJS Open..

[2] Qian C (2021). Gland Surg..

[3] Abrahimi MS (2021). Int J Environ Res Public Health..

[4] Albornoz CR (2015). Plast Reconstr Surg..

[5] Chen Z (2015). J Cancer Res Ther..

[6] Silva E (2014). Breast J..

[7] Luo Y (2024). Breast Cancer..

[8] Vicini FA (2019). Lancet..

[9] Bartelink H (2015). Lancet Oncol..

[10] Goyal A (2021). BMJ Open..

[11] Coles CE (2017). Lancet..

[12] Kim H (2021). Ann Surg Oncol..

[13] Yu T (2022). Breast J..

